# Microfluidics for adaptation of microorganisms to stress: design and application

**DOI:** 10.1007/s00253-024-13011-x

**Published:** 2024-01-22

**Authors:** Ahmed E. Zoheir, Camilla Stolle, Kersten S. Rabe

**Affiliations:** 1 Department of Genetics and Cytology, Biotechnology Research Institute, National Research Centre (NRC), 33 El Buhouth St., Dokki, Cairo, 12622 Egypt; 2 Institute for Biological Interfaces 1 (IBG-1), Biomolecular Micro- and Nanostructures, Karlsruhe Institute of Technology (KIT), Hermann-von-Helmholtz-Platz 1, 76344 Eggenstein-Leopoldshafen, Germany

**Keywords:** Microfluidics, Adaptive laboratory evolution, Microbial adaptation, Gradient systems, Stress resistance, Strain improvement

## Abstract

**Abstract:**

Microfluidic systems have fundamentally transformed the realm of adaptive laboratory evolution (ALE) for microorganisms by offering unparalleled control over environmental conditions, thereby optimizing mutant generation and desired trait selection. This review summarizes the substantial influence of microfluidic technologies and their design paradigms on microbial adaptation, with a primary focus on leveraging spatial stressor concentration gradients to enhance microbial growth in challenging environments. Specifically, microfluidic platforms tailored for scaled-down ALE processes not only enable highly autonomous and precise setups but also incorporate novel functionalities. These capabilities encompass fostering the growth of biofilms alongside planktonic cells, refining selection gradient profiles, and simulating adaptation dynamics akin to natural habitats. The integration of these aspects enables shaping phenotypes under pressure, presenting an unprecedented avenue for developing robust, stress-resistant strains, a feat not easily attainable using conventional ALE setups. The versatility of these microfluidic systems is not limited to fundamental research but also offers promising applications in various areas of stress resistance. As microfluidic technologies continue to evolve and merge with cutting-edge methodologies, they possess the potential not only to redefine the landscape of microbial adaptation studies but also to expedite advancements in various biotechnological areas.

**Key points:**

*• Microfluidics enable precise microbial adaptation in controlled gradients.*

*• Microfluidic ALE offers insights into stress resistance and distinguishes between resistance and persistence.*

*• Integration of adaptation-influencing factors in microfluidic setups facilitates efficient generation of stress-resistant strains.*

## Introduction

**Fig. 1:**
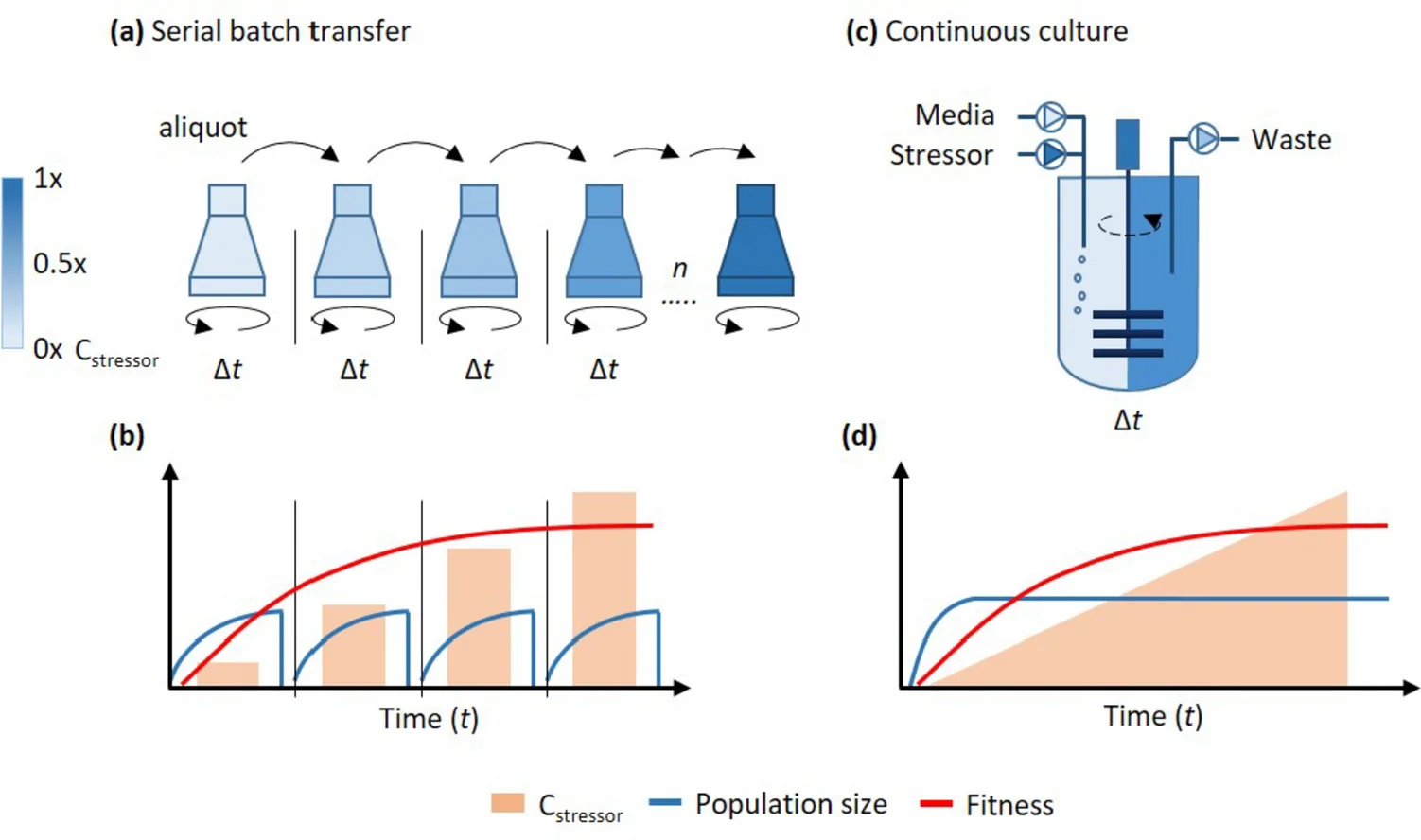
General concept of main adaptive laboratory evolution (ALE) approaches. **a** ALE through batch culture. Aliquots are serially transferred at regular time intervals (Δ*t*) to new cultures with a gradually increased stressor concentration. **b** In this system, population size is dynamically changing in every batch; however, the overall fitness is enhanced over time (*t*) as a result of the increasing stressor concentration. **c** ALE through continuous culture. Fresh media combined with a gradually increasing stressor concentration are fed continuously, and a proportional volume is removed to the waste. **d** Because of the optimized cultivation conditions, the population size mostly stays constant, while the overall fitness increases over time (*t*)

A key characteristic of living systems is their ability to interact with and respond to various chemical, physical, and biological factors in their environment. When these interactions have a detrimental effect, they are considered stressors, which can lead to reduced growth rates or compromised survival (Vorob'eva 2004). Sudden environmental changes can have a fatal impact on cells, with survival favoring those already genetically equipped to withstand the stress. In contrast, when changes occur gradually, cells can employ sophisticated molecular mechanisms to sense and adapt to specific stress factors through temporal metabolic adjustments or permanent genetic alterations (Brooks et al. 2011; Foster 2007; Galhardo et al. 2007; Zoheir et al. 2023). When such adaptations result in an inherited fitness advantage for a cell population, this phenomenon can be referred to as “adaptive evolution” (Rosenberg 2001) or simply “adaptation.” In this mini-review, we will summarize design concepts and applications of microfluidic systems for the study of adaptation of microorganisms to stress. The ability of cells to spontaneously adapt to different various physicochemical environments (Van den Bergh et al. 2018) can be harnessed to deliberately induce microbes to acquire novel traits or enhance their existing characteristics. This process is termed adaptive laboratory evolution (ALE) (Dragosits and Mattanovich 2013; Lässig et al. 2023; Portnoy et al. 2011; Wang et al. 2022; Wu et al. 2022). ALE is an essential methodology for investigating a variety of fundamental questions, encompassing the evolution of life, its underlying mechanisms, and the adaptive responses of microbial populations to their environments, including the development of antibiotic resistance (Card et al. 2021; Jahn et al. 2017; Lässig et al. 2023; Lázár et al. 2013; McDonald 2019; Stevanovic et al. 2022; Van den Bergh et al. 2018). Especially in the context of biotechnological production, ALE has found application in the enhancement of yields and the augmentation of an organism’s resistance to adverse conditions (Dragosits and Mattanovich 2013; Portnoy et al. 2011). Although ALE has numerous applications, it is fundamentally grounded in the principles of biological evolution, which encompass two interconnected processes: genetic variation and selection (Lässig et al. 2023; Van den Bergh et al. 2018). Genetic mutations naturally accumulate during DNA replication as spontaneous, random, and infrequent events, but their incidence can be increased in response to stress or other external factors (Matic 2017; Van den Bergh et al. 2018). Some of these mutations can confer the ability for microorganisms to thrive in typically inhibitory conditions, enhance their ability to consume specific substrates, or increase their efficiency in converting certain compounds. Selective retention of such advantageous traits is the primary objective of ALE. Therefore, an effective ALE technique should combine a high degree of genetic diversification with a well-defined strategy for selecting improved variants, which can be achieved through either batch or continuous cultures (Fig. 1). Especially when coupled with next-generation sequencing (NGS) and computational analysis tools, the process of discovering and mapping previously unknown mutations and their respective functions can be revolutionized (Fares 2015; Hirasawa and Maeda 2023). Furthermore, combining ALE with high-throughput methods such as microfluidic droplet screening enables the rapid improvement of relevant industrial producer strains (Chen et al. 2018; Luu et al. 2023; Weng et al. 2022; Yuan et al. 2022; Zhang et al. 2021).

From a technical standpoint, the most straightforward approach in using ALE for stress adaptation employs traditional cultivation equipment, such as shake flasks, for the sequential passaging of batch cultures over an extended duration while incrementally raising the selection pressure (Fig. 1a, b) (Mozhayskiy and Tagkopoulos 2013). This method enables the continuous selection of populations demonstrating improved fitness to specific environmental stresses (Fig. 1b). Historically, this method has proven successful in the adaptation of a variety of traits (Richard and Silver 1969) such as antibiotic resistance (Hoeksema et al. 2019; Jahn et al. 2017; Tirumalai et al. 2019) and a variety of microorganisms such as *Escherichia coli* (LaCroix et al. 2015), *Corynebacterium glutamicum* (Pfeifer et al. 2017), *Saccharomyces cerevisiae* (Hong et al. 2011), and *Chlamydomonas reinhardtii* (Yu et al. 2013).

Despite the simplicity and cost-effectiveness of these classical serial transfer methods, they are not without drawbacks. Notably, they entail labor-intensive manual culture manipulations on a daily basis, often spanning several months (Dragosits and Mattanovich 2013). To mitigate this challenge, researchers have explored the automation of ALE through the utilization of liquid-handling robots (Horinouchi et al. 2014), conventional chemostat bioreactors (Wallace-Salinas and Gorwa-Grauslund 2013), and customized cultivation devices (de Crécy et al. 2007; Wong et al. 2018) which serve to reduce the manual involvement and hands-on time required for the process. In contrast to serial transfer methods, a chemostat bioreactor offers the capability to maintain a continuous culture under precisely controlled growth, nutrient, and stress conditions (Fig. 1c, d) (Gresham and Dunham 2014). Typically, cultures are continually cultivated within an agitated vessel and supplied with fresh medium to sustain their exponential growth phase (Gresham and Dunham 2014; Jeong et al. 2016). This design enables the gradual application of stressors at defined concentrations, adjusting them over time in response to observed changes in growth fitness (Fig. 1d) (Jeong et al. 2016). Equipped with integrated sensors, this approach permits the automated regulation of various parameters, including culture density, pH, dissolved oxygen, and temperature (Gresham and Dunham 2014; Jeong et al. 2016). Researchers have harnessed this ALE strategy not only to bolster traits such as substrate utilization (Rajaraman et al. 2016) and tolerance to growth inhibitors (Koppram et al. 2012) but also to enhance resistance to antibiotic resistances (Chen et al. 2020; Fleming et al. 2002; Liu et al. 2016; Tonoyan et al. 2019). Notably, the characteristic of both batch and continuous culture systems is that they rely on vigorous mixing and thus are primarily suitable for the cultivation of planktonic populations. These systems also permit the gradual introduction of stressors over time. However, in natural environments, microorganisms often inhabit microenvironments characterized by heterogeneity and spatial gradients. These natural settings host a diverse mix of both planktonic and biofilm populations (Serra and Hengge 2014; Stewart and Franklin 2008). To simulate evolutionary processes in such natural conditions, alternative methodologies have been developed. For example, Baym et al. (2016) introduced an innovative experimental design, known as the MEGA-plate (Microbial Evolution and Growth Arena), which enables the study of adaptation to antibiotic resistance within spatial gradients. This setup allows for the observation and tracking of evolutionary dynamics in nonhomogeneous populations across spatially heterogeneous stress landscapes. Nonetheless, limitations such as plate size, contamination concerns, and its applicability only to motile strains restrict its use to specific applications. Therefore, there is an ongoing demand for alternative ALE technical systems that provide accessibility to the less-explored aspects of natural settings of adaptation.

## Design considerations when using microfluidics for microbial cultivation and ALE

Recent research has emphasized the significance of concentration gradients, biofilm communities, and heterogeneous microenvironments in influencing the composition and adaptability of bacterial populations (Baym et al. 2016; Coenye et al. 2022; Frost et al. 2018; Hermsen et al. 2012; Nagy et al. 2018). Yet, the incorporation of spatial chemical gradients (Baym et al. 2016) into meso- and macroscopic ALE systems presents notable technical challenges. Consequently, the use of miniaturized fluidic chip systems has gained increasing prominence in the study of microbial systems (Burmeister et al. 2018; Gucluer and Guler 2023; Hansen et al. 2019; Huang et al. 2023; Li et al. 2023; Ma et al. 2020; Matilla 2022; Pérez-Rodríguez et al. 2022; Täuber et al. 2021; Weibel et al. 2007), particularly those involving integrated concentration gradients for adaptive evolution (Deng et al. 2019; Nagy et al. 2022; Stevanovic et al. 2022; Zhang et al. 2011a). Thus, investigating adaptation within a miniaturized fluidic system emerges as a compelling design strategy.

The advent of microfabrication technologies, particularly microfluidics, has brought about a paradigm shift in contemporary biological research in recent years (Banik et al. 2023; Dai et al. 2023; Duncombe et al. 2015). Generally, microfluidics pertains to the principles and instrumentation for the manipulation and analysis of fluids on a micrometer scale (Beebe et al. 2002). It encompasses the utilization of singular or multiple microchannels, which can be intricately interconnected to establish networks or fluid trajectory patterns, and may incorporate specialized components such as mixers, valves, or electrodes for precise fluid control within the system. The miniaturization of experiments through microfluidics results in reduced liquid volumes, thereby reducing the consumption of costly chemicals and reagents, thus enhancing the cost-effectiveness of experiments. A crucial advantage of microfluidics lies in the concept of integration, where diverse functions, reactions, and processes can be seamlessly incorporated within the same platform, a concept commonly referred to as “lab-on-a-chip” (Mark et al. 2010; Streets and Huang 2013). Through integration, experiments can be designed and executed in a manner that may be challenging or even infeasible within traditional laboratory settings (Mark et al. 2010; Streets and Huang 2013; Täuber et al. 2021). Focusing on their utilization with microbial systems and given that microorganisms naturally inhabit microscale environments, microfluidic technologies intrinsically provide an invaluable platform for the cultivation and study of microorganisms under well-defined, custom-tailored microenvironments.

The fabrication of such microfluidic systems can be realized through utilizing various advanced technologies, including fused deposition modeling, soft lithography, micro-milling, and 3D printing (Gale et al. 2018; Grösche et al. 2019; Silverio and Cardoso de Freitas 2018; Zhang et al. 2022). Given the unavailability of high-end microfabrication technologies in most microbiology laboratories, researchers have devised affordable do-it-yourself (DIY) approaches to rapidly prototype and construct microfluidic systems tailored for biological research (Shin and Choi 2021; Tiwari et al. 2020). With such accessibility and rabid developments in the field, microfluidics have found widespread applications across diverse domains in microbiology and biotechnology in general (Hirasawa and Maeda 2023; Ortseifen et al. 2020; Saleh-Lakha and Trevors 2010; Scheler et al. 2019) and in aspects that can be harnessed for ALE in particular. For example, the development of microfluidic platforms has notably benefited the study of microbial biofilms, which are defined as aggregates of microorganisms enclosed within a self-produced matrix of extracellular polymeric substance (EPS), resulting in adhesion to each other and/or a surface (Vert et al. 2012). It is essential to note that biofilms represent the prevailing microbial structure and exhibit distinct physiological and gene expression profiles in comparison to planktonic cells (Flemming and Wuertz 2019; Stoodley et al. 2002), thus expected to play a fundamental rule in adaptation. Microfluidic systems have significantly advanced the examination of biofilm communities under controlled liquid flow conditions, enabling investigations into biofilm formation and adhesion mechanisms (Alles and Rosenhahn 2015; Kim et al. 2012; Straub et al. 2020), response to stressors such as antibiotics (Coenye et al. 2022; Dai et al. 2016; Kim et al. 2010; Zhou et al. 2021), and their application in biocatalysis (Halan et al. 2012; Hansen et al. 2019; Lemke et al. 2021; Willrodt et al. 2017) and microbial fuel cells (Choi 2015; Goel 2018). A comprehensive summary of microfluidic applications for biofilms can be found in the work by Pousti et al. (2019).

Besides biofilms, spatial concentration gradients are a common feature of microbial natural habitats (Dal Co et al. 2019), and their generation through microfluidic techniques involving flow mixing and chemical diffusion holds significant importance (Hu et al. 2017; Sweet et al. 2020). These methods have facilitated the investigation of microorganisms and their biofilms under controlled chemical gradients, serving various purposes such as the analysis of chemotaxis, toxicity assessment, and stress adaptation (Chung and Choo 2010; Deng et al. 2019; Li et al. 2014; Tang et al. 2022; Zhao and Ford 2022). Particularly in the context of stress adaptation, microfluidic devices featuring stable stressor gradients present a valuable means of miniaturizing ALE processes, where cells can be enriched at areas of low stress leading to accumulation of spontaneous mutations, whose only adapted traits can be selected for growth at areas of high stress. In such systems, microorganisms can gradually acclimate to increasing stressor concentrations, akin to the concept of the MEGA-plate (Baym et al. 2016). However, in contrast to the MEGA-plate, miniaturized chemical gradients involve the continuous provision of nutrients through fluid flow, mitigating growth inhibition resulting from nutrient limitations (Stevanovic et al. 2022).

**Fig. 2:**
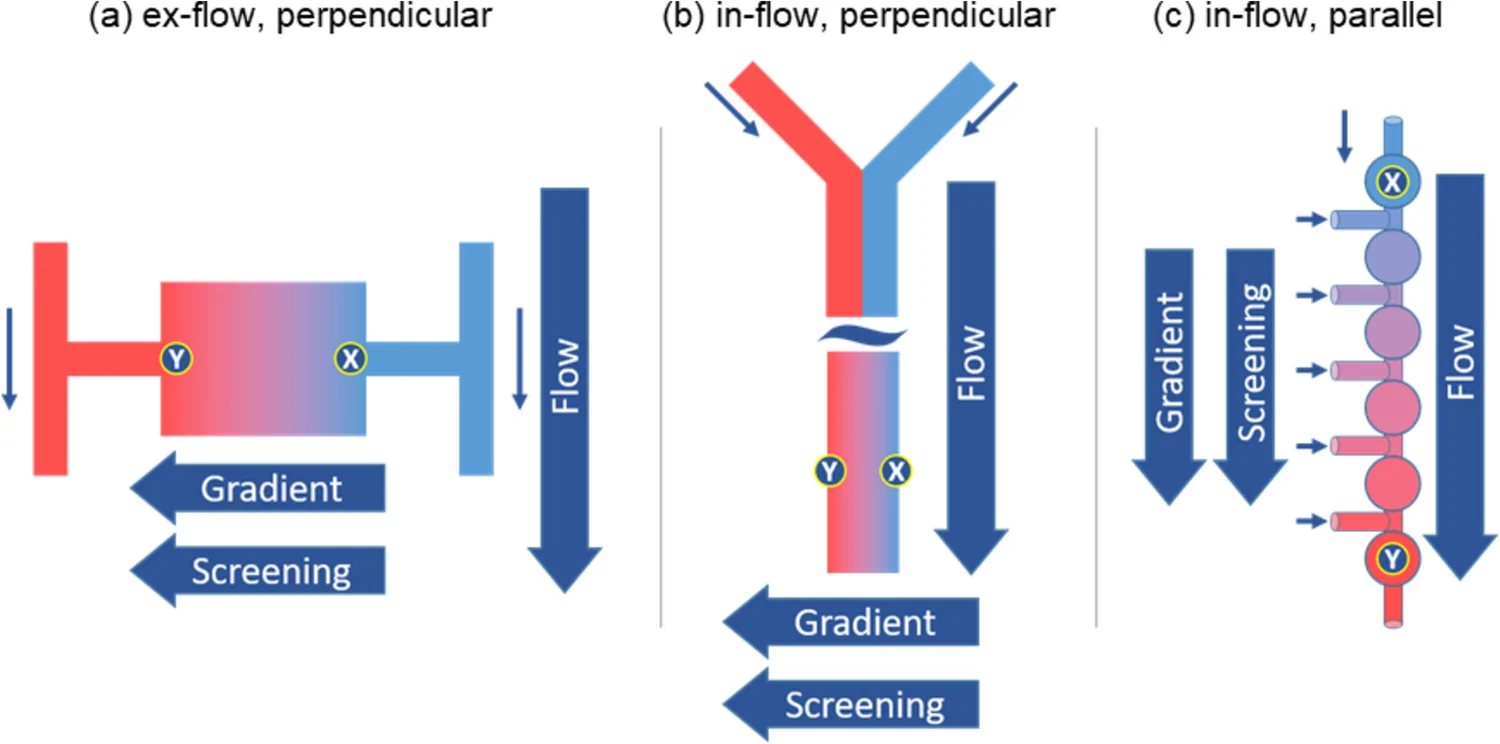
Commonly existing and alternative flow gradient creation strategies. The gradient chamber for cultivation may be positioned in two distinct configurations: external to the fluid flow, referred to as “ex-flow” (**a**), or internal to the fluid flow, denoted as “in-flow” (**b**, **c**) gradients. In the conventional ex-flow (**a**) and in-flow (**b**) systems, the gradient and screening processes are typically aligned perpendicularly with respect to the flow direction. In contrast, the alternative model introduced in (**c**) implements a parallel orientation of both the gradient and screening relative to the flow direction. Low-stress conditions are indicated in blue at point *X*, while high-stress conditions are depicted in red at point *Y*. The goal of adaptation is to move cells from the low-stress conditions (point *X*) for selection and to enrich cells that become adapted to stress at areas of high stress (point *Y*). Adapted with permission from Zoheir et al. (2021) and the Authors (2021) Small

With respect to gradient system design, the prevailing microfluidic gradient creation strategies employed in microbial studies can be categorized into two main approaches, according to the location of the gradient chamber relative to the flow direction: ex-flow and in-flow gradients (Fig. 2). In both ex-flow and in-flow models, microbial cultivation occurs within the gradient chamber, situated either outside or inside the flow, respectively (Choi et al. 2012; DiCicco and Neethirajan 2014; Hol et al. 2016; Irimia et al. 2006). In both models, the gradient typically positions perpendicularly relative to the flow direction (Fig. 2a, b), which may lead to suboptimal screening outcomes. In these systems, the screening and enrichment of adapted clones (corresponding to the movement of cells from point *X* to colonize at point *Y* in Fig. 2) primarily rely on biological forces, necessitating motile cells to actively traverse from regions of low stress (point *X*) toward zones with elevated stressor concentrations (point *Y*). This approach restricts the applicability of the system to motile bacteria. Moreover, bacterial motility is governed by chemotactic decisions, which generally lead cells away from high-stress environments (Deng et al. 2019; Gurung et al. 2020; Piskovsky and Oliveira 2023), thus hindering the screening. Particularly in the in-flow model (Fig. 2b), active cell movement can be further influenced by orthogonal flow forces. Consequently, in both gradient approaches, there exists a substantial risk that potentially adapted cells may never reach the high-stress areas of the microfluidic chip where the selection process takes place (point *Y*) (Deng et al. 2019).

Furthermore, prolonged cultivation of microorganisms in microfluidic chips, such as that used for adaptation experiments, can often clog the chip with accumulated biomass, blocking the delicate flow systems that generate the gradients. As a consequence, a majority of microbial investigations conducted within microfluidic gradients tend to focus on short-term cultivation (Diao et al. 2006; DiCicco and Neethirajan 2014; Hou et al. 2014; Liu et al. 2017; Zhang et al. 2011a). To surmount these challenges and facilitate extended microfluidic ALE experiments while maintaining effective screening under unfavorable high-stress conditions, an in-flow gradient system aligned with the flow direction has been recently developed (Fig. 2c) (Zoheir et al. 2021). Within this design configuration, the gradient chamber is positioned in-flow, parallel to the direction of the flow. The gradient chamber is further partitioned into discrete compartments that function as microenvironments, simulating natural niches that microorganisms favor. Furthermore, the screening under high-stress conditions in such a system is bolstered by flow forces propelling cells toward the up-gradient regions where stressor concentrations are elevated (point *Y*). Consequently, neither cellular motility nor chemotactic forces play a pivotal role, thus broadening the scope of potential strains available for adaptation.

## Applications of microfluidic systems for stress adaptation using ALE

**Fig. 3:**
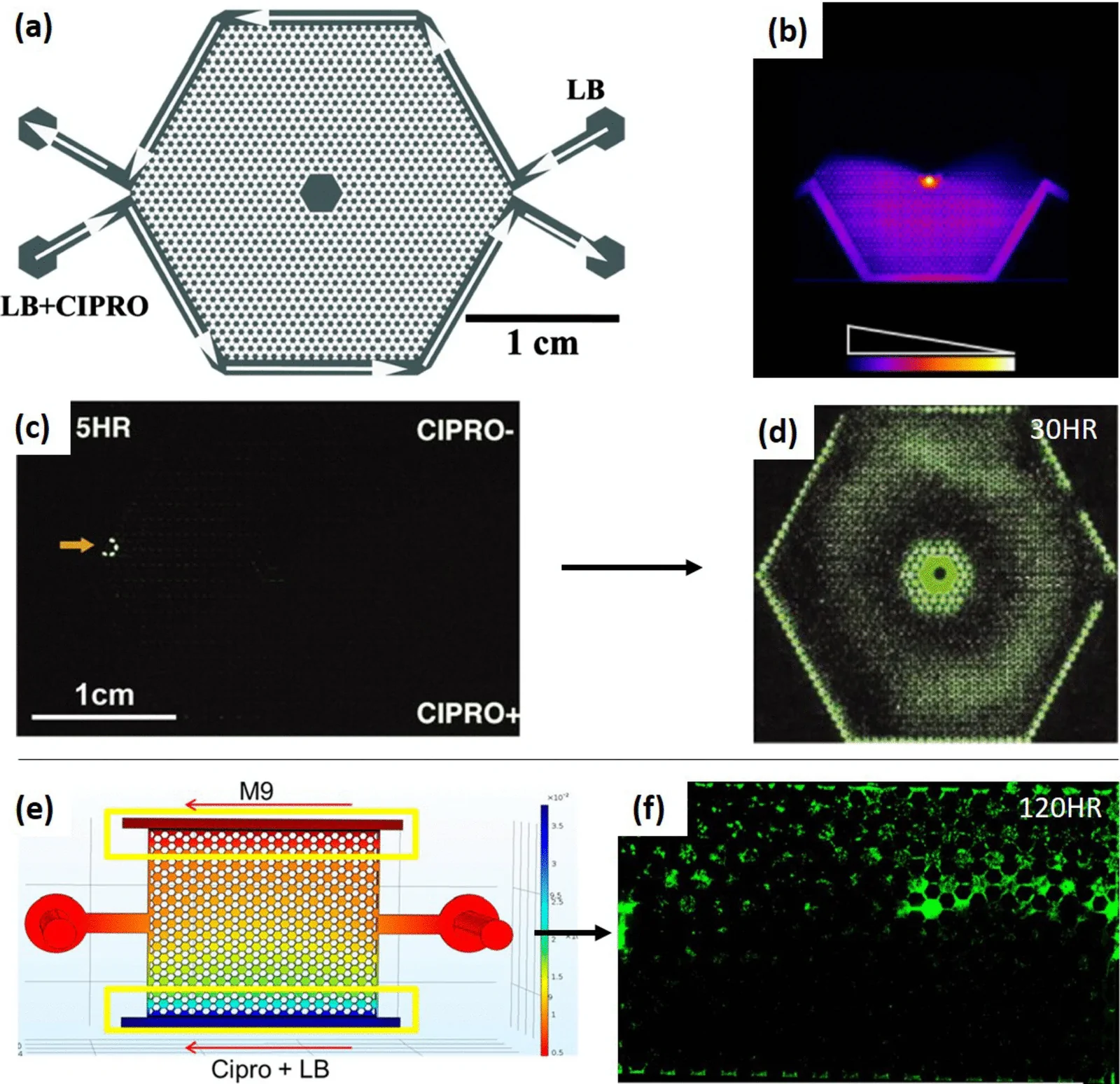
Microfluidic systems for adaptive laboratory evolution (ALE). **a**–**d** Hexagonal gradient chamber for ciprofloxacin adaptation (Zhang et al. 2011b). **a** This system comprises a network of interconnected microwells, designed for the adaptation of microorganisms to ciprofloxacin (Cipro) within LB medium. **b** A gradient of ciprofloxacin is meticulously established within the chamber, as depicted. **c** Remarkably, resistant *E. coli* cells emerge within a mere 5 h, particularly at the steepest gradient point, aptly referred to as “Goldilocks” (highlighted by the orange arrow). **d** Subsequently, the adapted cells manifest their growth, visualized through green fluorescence after 30 h. **e**, **f** Gradient system for stress-directed growth (Deng et al. 2019). **e** In this system, a unique gradient strategy is employed, focusing on directing microbial growth toward regions with high-stress levels. On one side of the chip, a M9 minimal medium with limited nutrients coexists with LB medium containing not only rich nutrients but also the antibiotic ciprofloxacin (Cipro). **f** *E. coli* growth is clearly marked by green fluorescence in this innovative gradient system. **a** Adapted with permission from Zhang et al. (2011b), copyright (2011) American Chemical Society. **b** Adapted with permission from Bos and Austin (2018), copyright (2018) Elsevier. **c**, **d** Adapted with permission from Zhang et al. (2011a), copyright (2011) The American Association for the Advancement of Science. **e**, **f** Adapted with permission from Deng et al. (2019), copyright (2019) American Chemical Society

As elucidated earlier, the comprehensive integration of microfluidic system designs embracing specific flow strategies aligning with adaptation, enrichment, and screening processes has demonstrated notable efficiency and significant promise. Subsequently, we will employ antibiotics as a paradigmatic model to underscore the mechanisms of resistance adaptation in microfluidic systems. A seminal illustration of stress adaptation using ALE conducted on microfluidic gradient landscapes of antibiotics was established by Zhang et al. 2011a, who devised an array of microwells designed to generate an ex-flow diffusion-based spatial gradient of ciprofloxacin for adaptation of *E. coli* (Fig. 3a–d). In this system, the hexagonal chip design (Fig. 3a) creates gradients between opposing sides of the chip (Fig. 3b). Notably, the initial appearance of ciprofloxacin-resistant mutants of *E. coli* was observed at the point with the steepest gradient, which has been coined as the “Goldilocks” point (Fig. 3c, denoted by the orange arrow). This phenomenon was attributed to the lower population of wild-type cells at this specific location, allowing mutant cells to swiftly establish themselves and proliferate (Zhang et al. 2011a). However, the gradient in this system can only be adjusted by altering the stressor concentration in the initial flow (Fig. 3a, LB + CIPRO), resulting in a predetermined gradient profile dictated by diffusion and lacking spatial customization. Furthermore, the fluid flows through the peripheral channels surrounding the growth chamber, constituting an ex-flow gradient, and thus exerts no physical influence on the cells. Consequently, screening within such a system necessitates the autonomous movement of cells through the gradient profile, thereby confining the utility of the system to motile strains exclusively. Moreover, due to inherent stress-sensing and chemotactic mechanisms, even motile cells generally exhibit a tendency to move away from stress, thereby undermining the efficacy of screening for potentially superior mutants. Nevertheless, this system demonstrated a remarkable capability to generate ciprofloxacin-resistant *E. coli* within a mere 10-h timeframe, starting with an initial inoculum as low as 100 bacteria. Clones were successfully identified, which demonstrated survival in LB media containing ciprofloxacin stressor concentrations up to 200 times the minimal inhibitory concentration (MIC).

To encourage the migration of cells toward regions with elevated stressor concentrations, strategies akin to those elucidated by Deng et al. (2019) can be employed. This strategy involves the incorporation of additional gradients of nutritional compositions (as illustrated in Fig. 3e, f) to generate regions characterized by both elevated stress levels and heightened nutrient concentrations, while concurrently establishing nutrient-poor zones in regions with low-stress levels. This approach is intended to attract cells from nutrient-poor, low-stress areas (Fig. 3e, M9) toward areas of nutrient-rich, high-stress for selection (Fig. 3e, Cipro + LB) (Deng et al. 2019). However, it is important to note that within such a setup, cell growth is fundamentally reduced at nutrient-poor areas, resulting in only modest levels of adaptation to ciprofloxacin at the selection zone, typically around twice the original MIC. Such a modest improvement of bacterial tolerance to stress underscores the challenges associated with expediting the ALE process when employing this particular strategy. It is worth emphasizing that such approaches, while innovative, often necessitate a trade-off between adaptation speed and the achievable level of adaptation, considering also the system compatibility with long-term adaptations. This requires careful consideration of the specific goals and constraints of the stress adaptation using ALE. While such strategies may lead to more thorough and precise adaptation, it may also extend the timeline for adaptation to reach desirable levels, requiring a stable system under prolonged experiments. Hence, it is essential to weigh these factors when selecting and implementing microfluidic systems for ALE.

**Fig. 4:**
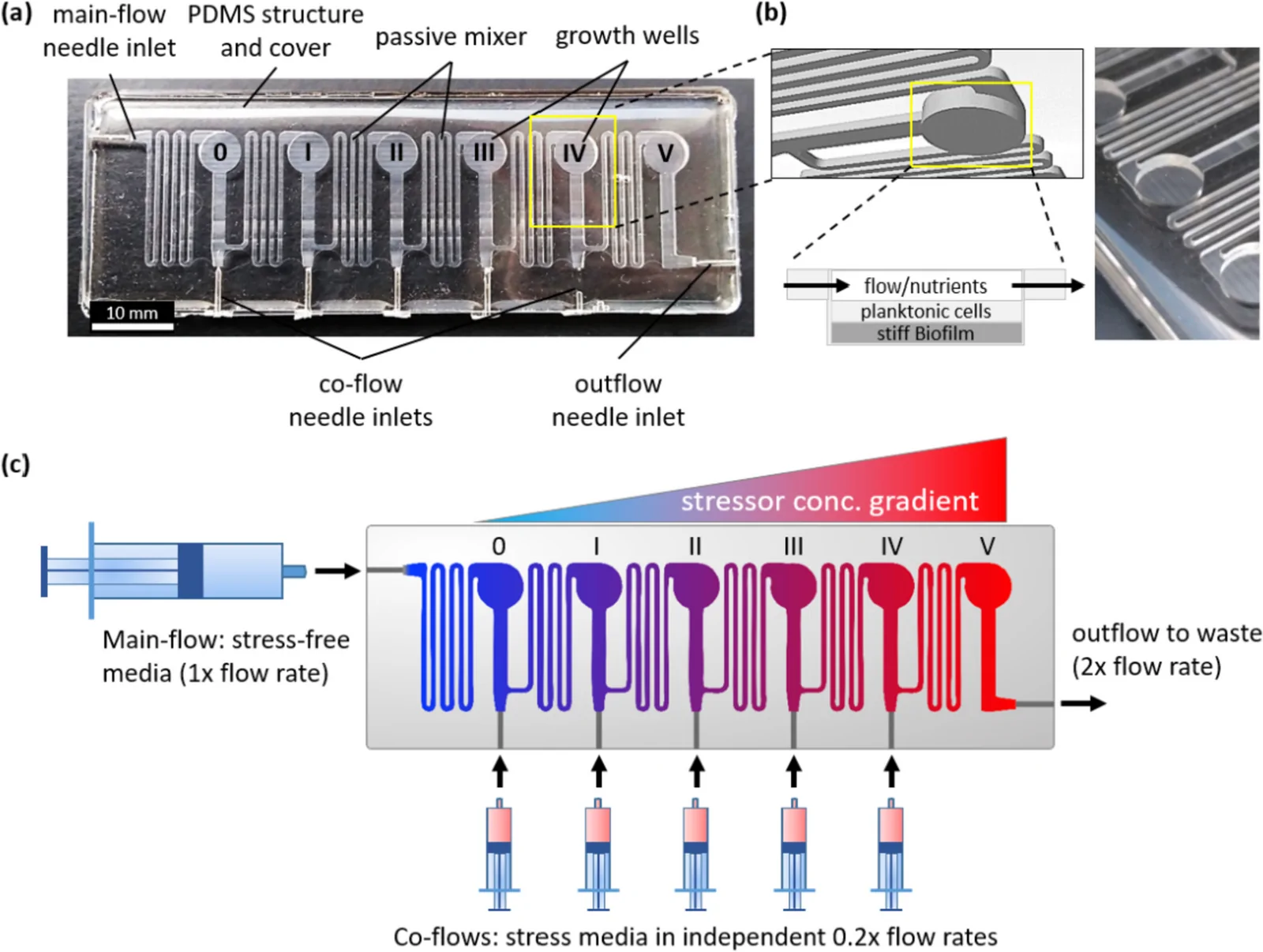
Structure of the evo.S microfluidic chip. **a** The evo.S chip is fabricated using polydimethylsiloxane (PDMS), a biocompatible and gas-permeable material that ensures an ideal environment for microbial growth. **b** The chip accommodates microbial cultures within wells that serve as versatile microenvironments for hosting mixed populations of planktonic cells and biofilms. The dimensions of the wells are carefully tailored, and the wells are designed to be deeper than the channels. **c** The evo.S chip’s distinctive feature lies in its capability to cumulatively create chemical gradients within interconnected wells. This is achieved through the stepwise supplementation of the main flow with defined stressor concentrations at precise flow rates. The controlled formation of gradients is a pivotal aspect of the chip’s functionality, contributing to the success of ALE experiments conducted within this system. Reprinted with permission from Zoheir et al. (2021) and the Authors (2021) Small

An alternative approach to enhance the robustness of ALE process including efficient screening of the generated cell populations, is to establish an inflow gradient aligned parallel to the flow direction, which incorporates an adjustable spatial stress gradient for the effective on-chip screening of the entire cell population with minimal trade-offs (Zoheir et al. 2021). This innovative concept, referred to as evo.S (short for *evo*lution under *s*tress), is realized within the chip through the creation of stepwise, cumulative increases in stressor concentrations across interconnected 3D compartments (Fig. 4). The evo.S chip is manufactured using polydimethylsiloxane (PDMS), a biocompatible and gas-permeable material (Fig. 4a). This design possesses the capacity to foster the growth of microorganisms in both planktonic and biofilm forms residing in these 3D microcompartments (Fig. 4b), offering a unique platform to investigate and harness the adaptive potential of microbial populations under controlled conditions. Furthermore, this system’s versatility, accommodating both planktonic and biofilm growth, widens the scope of ALE studies and facilitates a more comprehensive exploration of microbial adaptation strategies. Notably, the 3D compartments are interconnected through passive diffusion mixers, which aids in the homogenization of the gradient, contributing to consistently formed concentrations in the compartments.

**Fig. 5:**
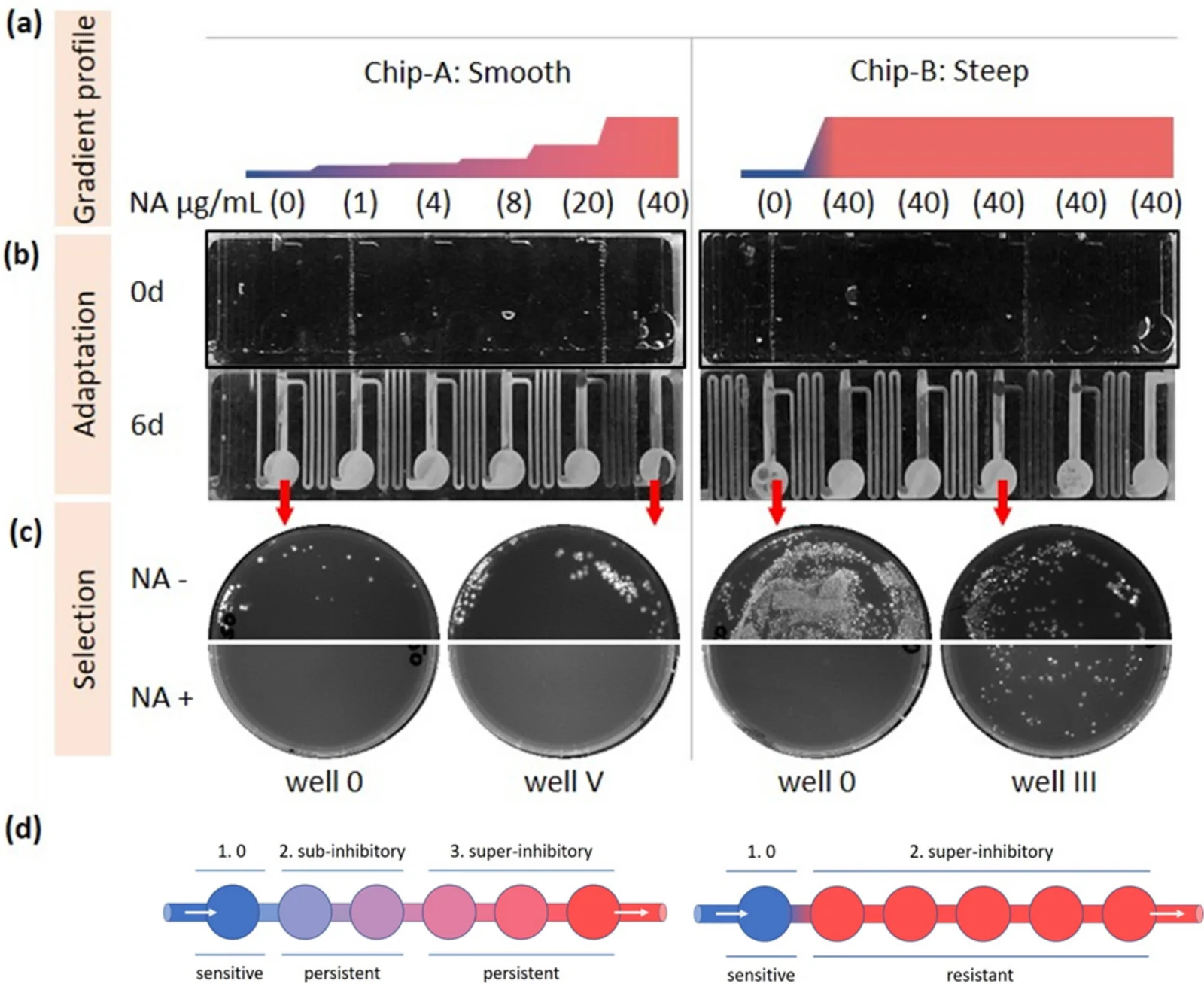
Impact of gradient profiles on the adaptability of *E. coli* to nalidixic acid (NA) within the evo.S chip. **a** The study employed two distinct concentration profile modes: a smooth gradient denoted as “chip-A” and a steep gradient designated as “chip-B.” Remarkably, both profiles yielded similar *E. coli* growth on the chip after approximately 6 days (**b**). **c** To assess the adaptability of *E. coli* populations, screening was conducted on selective agar plates containing 40 μg/mL NA. Notably, the colonies observed on these plates indicated successful evolution, primarily within the context of the steep gradient (chip-B) profiles, thus underscoring the role of gradient steepness in facilitating adaptation. **d** The influence of the antibiotic gradient on adaptation phenotypes within the evo.S chip. In the case of chip-A (smooth gradient), bacteria experience a sequential transition through phases: (1) a phase devoid of antibiotics, (2) a phase containing sub-inhibitory concentrations inducing persister cell formation, and (3) a phase featuring super-inhibitory concentrations. In this scenario, persister cells emerge and can endure the subsequent super-inhibitory concentrations, leading to persistence. In contrast, chip-B (steep gradient) directly exposes sensitive cells from phase (1) to super-inhibitory concentrations in phase (2), inducing and selecting for resistant mutants. Adapted with permission from Zoheir et al. (2021) and the Authors (2021) Small

To establish a stress gradient (as depicted in Fig. 4c), the methodology involves introducing stressor-free medium through the primary inlet (main flow), while utilizing the five side inlets (co-flows) to create a tailored spatial concentration gradient via a medium with precisely defined stressor concentrations and flow rates. Once the gradient attains stability under a continuous flow regime, a bacterial inoculum is introduced into the stressor-free well (labeled 0). Subsequently, the progeny cells are consistently flushed downstream by the force of fluid flow, directed toward wells I–V featuring higher stressor concentrations. Given the survival of only those clones possessing superior fitness under a given stress, a novel population takes residence within these wells only when it is equipped with the qualifying genotype to thrive at the respective stressor level, while concurrently all other clones those still susceptible to stress are washed out into waste. This unique approach, exemplified by the evo.S chip, was effectively employed for the evolution of *E. coli* resistant to the antibiotics nalidixic acid (NA) as well as rifampicin. What sets the evo.S chip apart is its ability to facilitate the cultivation of cells within a customizable concentration gradient, offering versatility in terms of gradient profiles, ranging from gradual (Fig. 5a, chip-A) to steep (Fig. 5a, chip-B). *E. coli* cells were subjected to cultivation within the respective NA gradients over a period of 6 days (Fig. 5b). Notably, there were no incidents of chip clogging throughout the course of the experiment, and upon conclusion, complete growth was observed in all wells of both chips. This observation underscores the successful adaptation of *E. coli* to the highest levels of NA toxicity.

Upon sampling of cells from the chip and transferring them on NA-selective plates, a distinctive pattern was unveiled, in which the steep gradient notably facilitated the emergence of *E. coli* mutants, whereas the smooth gradient did not yield any such mutants. This pattern was consistent and replicated with the antibiotic rifampicin, underscoring the chip’s capacity to induce adaptation in cells toward varying antibiotic resistances. Notably, this influence of gradients on the adaptation phenotype appears to be a recurring phenomenon in the context of *E. coli* adaptation to antibiotics, a facet that has thus far received limited detailed investigation. It is noteworthy that both gradient modes employ identical final antibiotic concentrations and exhibit full growth on the chip. These interesting observations may be attributed to the effects of sub-inhibitory doses of antibiotics (Fig. 5d), which are known to trigger bacterial stress responses (SR) (Andersson and Hughes 2014). In the context of the smooth gradient chip, bacterial cells encounter sub-inhibitory antibiotic concentrations in the initial wells. Under these conditions, two key phenomena may take place: the generation of persister cells and the formation of biofilms. These mechanisms can provide a protective shield to the population against antibiotics, resulting in a collective protection strategy, often referred to as “persistence” (Fux et al. 2005). In contrast, within the steep gradient chip, stress-induced mutagenesis (SIM) can occur. SIM represents a hyper-mutagenesis mechanism that can result in the development of genuine genetic resistance to antibiotics (Kohanski et al. 2010). These interesting findings emphasize the role of gradient profiles enabled by microfluidic systems in influencing the adaptive responses of *E. coli* to stressors exemplified by antibiotics, shedding light on the multifaceted mechanisms that govern microbial adaptation under varying stress conditions. Further research in this domain is warranted to unravel the nuances of these adaptive strategies and their broader implications in antibiotic resistance development.

“Persistence” and “resistance” represent distinct facets of microbial adaptation, with the former characterizing a transient and condition-specific phenotypic fitness and the latter denoting a stable genotypic alteration. The evo.S chip introduced a valuable capability to differentiate between persistence and resistance, which can enhance the comprehension of previously conducted studies, such as the work by Deng et al. (2019). As mentioned above, Deng et al. (2019) in their investigation have cultivated *E. coli* in a microfluidic array featuring a smooth gradient of ciprofloxacin over a 5-day period, yielding a relatively modest increase in resistance levels (approximately twice the original MIC), as compared to standard homogeneous batch cultivation employed as a control. The design of their system incorporated an ex-flow gradient, as elucidated in Fig. 2a and Fig. 3e, f, and the screening process relied on the unaided motility of cells from low- to high-stress regions. Their assumption was that cells did not demonstrate a preference for the far up-gradient areas, thus potentially failing to migrate toward regions characterized by elevated antibiotic concentrations. However, when examining the in-flow smooth gradient model employed by the evo.S chip (designated as “chip-A”), where cells are compelled by fluid flow to move toward regions up-gradient featuring higher antibiotic concentrations, cells have also demonstrated no adaptation. This suggests that the dynamics of cell chemotaxis and motility alone do not fully explain the relatively low frequency of adapted cells within the system studied by Deng et al. (2019) and that the gradient profile formed on the chip may have the biggest influence on the final adaptation behavior.

The significance of gradient steepness is further underscored when considering the alternate system detailed by Zhang et al. 2011a (Fig. 3a–d). As mentioned earlier, they made a notable observation wherein ciprofloxacin-resistant *E. coli* mutants emerged initially at the sites characterized by the steepest concentration increments “Goldilocks” (as indicated by the orange arrow in Fig. 3c). Intriguingly, this rapid adaptation occurred a mere 5 h after the introduction of 10^6^ cells (Zhang et al. 2011b). The fast adaptation observed in the context of the steep gradient resonates with the outcomes obtained from chip-B with the evo.S chip system (Zoheir et al. 2021), lending support to an “adapt-or-die” scenario. Two plausible explanations underlie this observed cell behavior. Firstly, it is feasible that motile wild-type cells may be present at the Goldilocks points on the chip but at low population densities. This scenario may allow *de novo* resistant mutants to rapidly establish themselves due to their heightened fitness in the face of elevated antibiotic concentrations (Frisch and Rosenberg 2011; Zhang et al. 2011a). Alternatively, the key factor in this phenomenon may not be the population density of wild-type cells but the pronounced steepness of the stress gradient. This characteristic could potentially drive swift adaptation in the Goldilocks areas through a mechanism known as SIM, as proposed by prior works (Frisch and Rosenberg 2011; Zhang et al. 2011a) and the findings of Zhang et al. 2011b. These contrasting possibilities offer valuable insights into the mechanisms underpinning the rapid adaptation of microbial populations to stressors in microfluidic systems and warrant further exploration in future research endeavors.

Adaptation to antibiotic as a manifestation to stress offers crucial insights for adapting industrial producer strains resilient to diverse environmental pressures. The mechanisms underlying microbial adaptation to antibiotics, observed and studied in controlled microfluidic environments, provide a blueprint for engineering industrial strains capable of withstanding stressors encountered in manufacturing processes and can improve the understanding how microorganisms develop resistance. This allows for targeted interventions to result in robust industrial producer strains that exhibit enhanced resilience and productivity amidst challenging production environments.

## Conclusion

In conclusion, in this mini-review, we have summarized how microfluidic systems can be exploited in the context of ALE to adapt microorganisms to stressors such as antibiotics. The intricate control over environmental conditions offered by microfluidics not only enhances our comprehension of stress responses but also opens avenues for engineering robust microbial strains. The integration of adaptation-influencing factors such as the steepness of chemical gradients, the contact time with a stressor, and the method of screening can enable precise tailoring of microbial phenotypes. As microfluidic technologies advance and converge with cutting-edge methodologies, the potential to redefine not just microbial adaptation studies but also to catalyze advancements across various biotechnological domains becomes increasingly apparent. In perspective, the integration of such microfluidic systems addressed above with automated liquid handling, machine-assisted culturing, droplet-based sorting (Kim et al. 2021; Zhu et al. 2019), and advanced analytical techniques (Hansen et al. 2019), coupled with the utilization of novel materials facilitating biofilm growth (Zoheir et al. 2022) and along with high-throughput genomics and transcriptomics (Reuter et al. 2015), represents a pivotal stride toward understanding the ground bases of molecular adaptation and also pushing the boundaries of developing enhanced industrial producer strains.

## Data Availability

All data and materials from Zoheir et al. (2021) are available from the authors upon reasonable request. For data and material from other literature, please contact the respective corresponding authors.
